# Genetic diversity and population structure of *Rheum tanguticum* (*Dahuang*) in China

**DOI:** 10.1186/1749-8546-9-26

**Published:** 2014-11-03

**Authors:** Xiaoqin Zhang, Ying Liu, Xuan Gu, Zhengzheng Guo, Li Li, Xiaona Song, Siqi Liu, Yimei Zang, Yanpeng Li, Chunsheng Liu, Shengli Wei

**Affiliations:** School of Chinese Pharmacy, Beijing University of Chinese Medicine, No. 6 Wangjing Zhonghuan South Street, Chaoyang District, Beijing, 100102 China; Lishui Hospital of Chinese Medicine, Zhejiang, 323000 China

## Abstract

**Background:**

Wild *Rheum tanguticum* (*Dahuang* in Chinese) has becoming endangered in China. This study aims to examine the genetic structure and genetic diversity of *R. tanguticum* within species, and the genetic differentiation within and among populations in China.

**Methods:**

The variability and structure of 19 populations of *R. tanguticum* were investigated by their chloroplast DNA *mat*K sequences. The genetic diversity index was calculated by Dnasp, PERMUT, and Arlequin 3.0 software, and a neighbor-joining (NJ)-tree was constructed by MEGA 5.0 software.

**Results:**

Fifteen haplotypes were obtained based on the *mat*K sequence analysis. The mean genetic diversity within species was 0.894, and the genetic variability among populations (67.6%) was relatively higher than that within populations (13.88%) according to the AMOVA and PERMUT analyses. The NJ-tree and a pairwise difference analysis indicated geographical isolation of *R. tanguticum*. The gene flow among populations was 0.05, indicating a genetic drift among some populations, which was also confirmed by the NJ-tree and haplotype distributions. Furthermore, a mismatch distribution analysis revealed the molecular evolution of *R. tanguticum*.

**Conclusion:**

Genetic diversity among and within populations of *R. tanguticum* in China was demonstrated.

## Background

*Rheum tanguticum* Maxim. ex Balf belongs to the family Polygonaceae, and grows mainly in high-altitude areas in the southwest and northwest of China, such as Sichuan, Gansu, and Qinghai provinces [1, 2]. The rhizomes and roots of *R. tanguticum* (*Dahuang* in Chinese) are used in Chinese medicine for unloading the tapping product, clearing *re* (heat), purging *huo* (fire), removing pathogenic *huo* from the *xue* (blood), stimulating menstrual flow, and promoting diuresis and detoxification [3–7]. The huge demand for *R. tanguticum* has caused excessive consumption in China [8–11]. The reproductive rate of *R. tanguticum* is low and environment-dependent, and the wild resources of *R. tanguticum* are becoming endangered [12].

Genetic diversity involves organism complexity [13], ecosystem recovery [14], and species sensitivity to environmental changes [15]. A lack of diversity reflected evidence for potential population endangerment [16, 17]. Various molecular markers were used to investigate the genetic diversity of *R. tanguticum*. Chen *et al.* [18] discovered a relatively high genetic diversity at the species level and a low genetic diversity within populations of *R. tanguticum* by evaluating an SSR marker. These findings were in accordance with those of Wang *et al.* [19] based on an ISSR marker. However, Hu *et al.* [20] demonstrated a similar result at the species level, but an opposite result within and among populations of *R. tanguticum* using an ISSR marker. These studies of *R. tanguticum* genetic diversity involved limited materials, and their results were contradictory. Therefore, large samples and new molecular markers were required to reveal the real state of *R. tanguticum* genetic diversity*.*

The *mat*K gene (1500 bp) is a molecular marker for plant molecular systematics and evolution, and is located within the intron of the chloroplast gene *trn*K on the large single-copy section adjacent to the inverted repeat [21]. Among various other molecular markers, the *mat*K gene sequence avoided any interference of heterozygosity and its evolutionary rate was relatively fast [22, 23]. Therefore, in recent years, the *mat*K gene has been employed as an important and powerful tool for examining intergenus and intragenus genetic diversity because of its high substitution rate [24, 25].

This study aims to examine the genetic structure and genetic diversity of *R. tanguticum* within species, and the genetic differentiation within and among populations in China. The genetic diversity of *R. tanguticum* at the species level and within and among populations was investigated using the *mat*K gene sequences, and the population structure of *R. tanguticum* was clarified.

## Methods

### Plant materials

**Figure 1:**
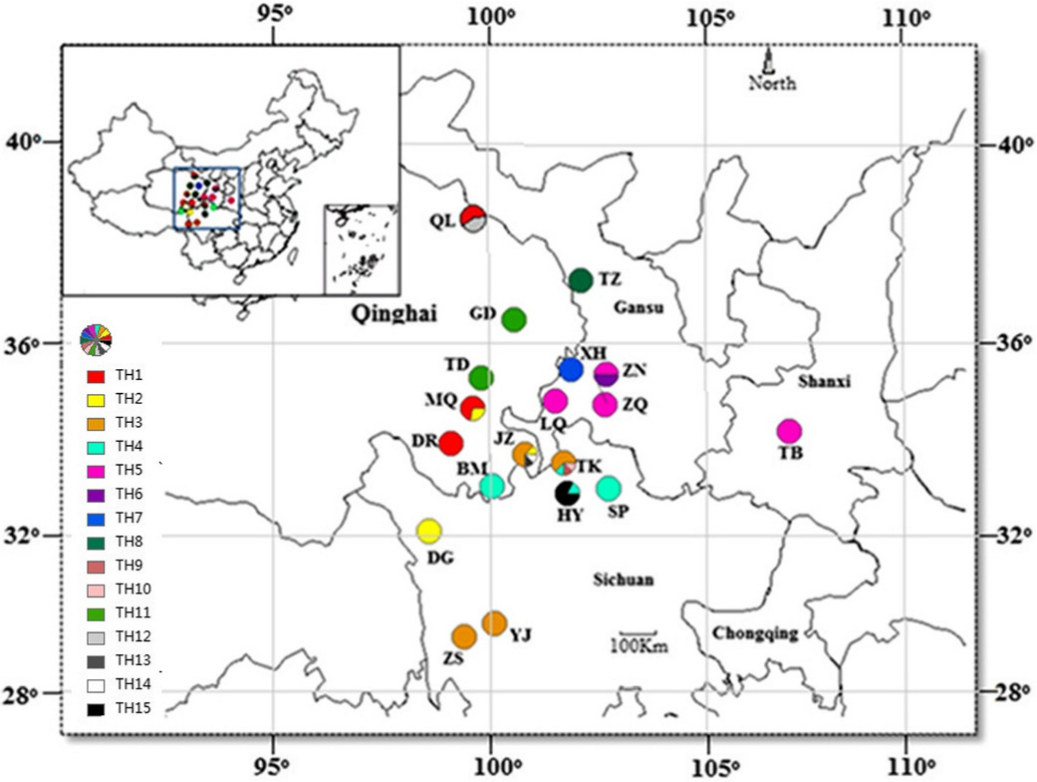
**Geographic distributions of the 19 populations and 15 haplotypes.** The pie chart shows the proportions of haplotypes in each population. The haplotype information was listed in Table 2.

A total of 276 *R. tanguticum* individuals were collected from 19 populations in Sichuan, Gansu, and Qinghai provinces of China (Figure 1). Each population was composed of 10–20 individuals spaced 50 m apart from one another. Tender leaves of each sample were stored in ziplock bags with silica gel. The latitude, longitude, and altitude of each collection site were recorded by an Etrex GIS unit (Garmin, Taiwan). The sample information is listed in Table 1.

**Table 1:** **The 19 populations of** ***R. tanguticum*** **and thei haplotypes (TH1–TH15) based on the** ***mat*** **K gene sequences**

Code	Locality	Altitude(m)	Number of samples	Haplotypes	Hd	Pi
BM	Banma,Qinghai	3694	20	TH4(20)	0	0
DR	Dari,Qinghai	3981	21	TH1(21)	0	0
MQ	Maqin,Qinghai	3746	21	TH1(15),TH2(6)	0.476	0.00063
GD	Guide,Qinghai	3728	12	TH11(12)	0	0
QL	Qilian,Qinghai	2981	18	TH1(10),TH12(8)	0.523	0.00276
JZ	Jiuzhi,Qinghai	3649	8	TH2(1),TH3(5),TH13(1),TH14(1)	0.643	0.00144
TD	Tongde,Qinghai	3728	20	TH11(20)	0	0
DG	Dege,Sichuan	3934	20	TH2(20)	0	0
HY	Hongyuan,Sichuan	3492	12	TH4(2),TH15(10)	0.333	0.00022
SP	Songpan,Sichuan	3282	10	TH4(10)	0	0
TK	Tangke,Sichuan	3447	8	TH3(5),TH4(1),TH9(1),TH10(1)	0.643	0.00115
ZS	Zhuosang,Sichuan	2700	10	TH3(10)	0	0
YJ	Yajing,Sichuan	4122	21	TH3(21)	0	0
XH	Xiahe,Gansu	3360	20	TH(20)	0	0
TB	Taibai,Shanxi	2833	21	TH(21)	0	0
TZ	Tianzhu,Gansu	3098	22	TH(22)	0	0
ZN	Zhuoni,Gansu	3558	8	TH5(4),TH6(4)	0.667	0.0022
ZQ	Zhouqu,Gansu	3000	10	TH5(10)	0	0
LQ	Luqu,Gansu	3233	12	TH5(12)	0	0

Hd: haplotype diversity; Pi: nucleotide diversity. The haplotype information is listed in Table 2.

### DNA extraction, PCR amplification, and sequencing

Total DNA was extracted from the silica gel-dried leaves using the CTAB method [26]. The *mat*K region was amplified with three pairs of primers. The first primer pair was *trn*K1895F (5′-GACATCCCATTAGTAAGCC-3′) and *trn*K2R (5′-AACTAGTCGGATGGAGTAG-3′), the second primer pair was *mat*kK592F (5′-TCCTACCGTGTGTGAATGCG-3′) and *mat*K8R (5′-AAAGTTCTAGCACAAGAAAGTCGA-3′), and the third primer pair was Pt-*trn*K692F (5′-GACTGTATCGCACTATGTATC-3′) and *trn*K1544R (5′-GGATAACCCCAGAATGCTTAG-3′). All primers were synthesized by Shanghai Shenggong Company (China). Each PCR amplification was performed in a 50-μL reaction mixture by a Cycler™ Thermal Cycler (Bio-Rad, USA) PCR procedure as follows: 94°C for 5 min; 35 cycles of 94°C for 45 s, annealing at 51°C for 1 min, and extension at 72°C for 1 min; final extension at 72°C for 10 min. A 1/10 volume of each PCR product was examined by electrophoresis in a 1.0% (w/v) agarose gel, and the remaining part was sequenced for correction.

### Data analysis

Sequences were aligned by ClustalX [27] and manually adjusted by BioEdit v.7.0.9 [28]. All gaps were treated as missing characters. Dnasp 4.0 estimated the molecular diversity, including the number of segregating sites (S), number of haplotypes (Nh), haplotype diversity (Hd), and nucleotide diversity (Pi) [29]. The Dnasp 4.0 also performed Tajima’s test and calculated the mismatch distributions [30]. PERMUT calculated the average gene diversity within populations (Hs), total gene diversity (Ht), and two measures of population differentiation, GST and NST (equivalent coefficient taking into account sequence similarities among haplotypes) [31]. Arlequin 3.0 software performed an analysis of molecular variance (AMOVA) to analyze the pairwise differences among and within populations [32]. The DNA divergences among populations (Fst) were measured, and the significances were tested using 10,000 permutations [33]. Gene flow between pairs of populations was calculated based on the Fst values (Nm = (1–Fst)/4 Fst). Statistical Product and Service Solutions (SPSS) calculated the correlation between genetic difference and geographic distance. A molecular phylogenetic tree was constructed by the neighbor-joining (NJ) method in MEGA 5.0, based on 87 samples including all of the haplotypes [34]. Insertions and deletions of base pairs were removed by the bootstrap method with 1000 replicates.

## Results

### Haplotypes and their distribution analysis

Among the 19 populations, a 1518-bp *mat*K sequence was obtained from 18 populations. The only exception was the TZ population from Gansu province, which produced a 1524-bp *mat*K sequence with a ‘TAAACC’ insertion at the 1022-bp site. A total of 21 segregated sites were found in the *mat*K sequence of *R. tanguticum*, and 15 haplotypes were determined (Table 2). There was only one haplotype in 13 populations, two different haplotypes in four populations, and four different haplotypes in the JZ and TK populations (Figure 1, Table 1). Among the 15 haplotypes, three haplotypes, TH3, TH4, and TH5, were simultaneously detected in four different populations. Two haplotypes, TH1 and TH2, were simultaneously detected in three different populations. TH11 was detected in two populations at the same time. The other nine haplotypes, TH6, TH7, TH8, TH9, TH10, TH12, TH13, TH14, and TH15, were only detected in one population.

**Table 2:** **Variable sites in the** ***mat*** **K gene sequences of the 15** ***R. tanguticum*** **haplotypes**

SNP																						
Haplotype	30	106	367	443	619	743	764	769	793	803	859	883	937	1022	1055	1106	1108	1117	1156	1267	1410	GenBank No.
TH1	A	G	C	T	A	C	A	T	G	T	C	C	C	C	C	T	C	G	C	A	T	KF880247
TH2	*	*	*	*	*	*	*	*	*	*	A	*	*	*	*	*	*	*	*	*	*	KF880035
TH3	*	*	*	A	*	A	*	*	*	*	*	*	*	*	*	*	*	*	*	*	*	KF880114
TH4	*	*	*	*	*	*	T	*	*	*	*	*	*	*	*	*	*	*	*	*	*	KF880006
TH5	*	*	*	A	C	A	*	G	*	*	*	*	*	*	*	*	*	*	*	*	*	KF880160
TH6	*	*	*	*	*	*	*	*	A	*	*	*	*	*	*	*	*	*	*	*	*	KF880104
TH7	*	*	*	A	*	A	*	G	*	*	A	*	T	*	*	*	T	*	*	*	G	KF880127
TH8	*	A	T	A	*	A	*	G	*	A	*	*	T	#	*	*	T	A	*	*	G	KF879968
TH9	*	*	*	A	C	A	*	G	*	*	*	*	*	*	*	*	*	*	*	G	*	KF879969
TH10	*	*	*	A	*	A	*	*	*	*	*	*	*	*	*	*	*	*	T	*	*	KF879972
TH11	*	*	T	A	*	A	*	G	*	A	*	*	T	*	*	*	T	*	*	*	G	KF879978
TH12	G	*	T	A	*	A	*	G	*	A	*	*	T	*	*	T	*	*	*	*	G	KF880023
TH13	*	*	*	A	*	*	T	*	*	*	*	*	*	*	*	*	*	*	*	*	*	KF880032
TH14	*	*	*	A	C	A	*	G	*	*	*	A	*	*	T	*	*	*	*	*	*	KF880033
TH15	*	*	*	*	*	*	*	*	*	*	*	*	*	*	*	*	*	*	*	*	*	KF880051

#: TAAACC. An asterisk indicates that the character states are the same as TH1.

### Genetic diversity analysis

The genetic diversity of the *mat*K sequences was relatively low in the same population, but relatively high in different populations (Table 1, Figure 1). The highest genetic diversity was observed in population ZN (Hd = 0.667, Pi = 0.0022), while the lowest genetic diversity was observed in 13 populations, *e.g.*, TH4 (Hd = 0, Pi = 0). The changes in Pi showed a similar trend toward haplotype diversity, and the only difference was that the highest Pi was found in population QL (Pi = 0.00276), rather than population ZN (Pi = 0.0022). The Hd and Pi values within the species were 0.894 and 0.00308, respectively, demonstrating a relatively high level of genetic diversity.

### Genetic differentiation and genetic difference analysis

The AMOVA results showed high variability among the populations (Table 3). The genetic differentiation among and within populations was 67.6% (FST = 0.82996) and 13.88% (FSC = 0.86121), respectively. The genetic differentiation was mainly observed among populations. According to the results of the PERMUT analysis, the genetic diversity among populations (Ht = 0.918) was higher than that within populations (Hs = 0.173), which was consistent with the AMOVA results. The value of NST (0.854) was higher than the value of GST (0.812), indicating a differentiation of geographical structure among populations of *R. tanguticum*.

**Table 3:** **Analysis of molecular variance (AMOVA) results for all haplotypes**

Source of variation	d.f.	SSD	Variance component	Percentage of variation	F-statistics	***P***value
Among groups	2	96.13	0.5026	18.52	FCT = 0.18523	=0.056
Among populations	16	264.65	1.83423	67.6	FST = 0.82996	<0.001*
Within populations	154	57.996	0.3766	13.88	FSC = 0.86121	<0.001*
Total	172	172	2.71343	-	-	-

d.f.: degrees of freedom; SSD: sum of squares. *Significance values after 1000 permutations.

**Figure 2:**
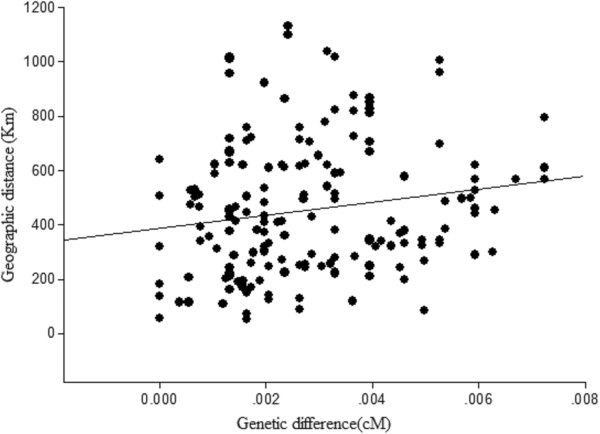
**SPSS analysis results for the correlation between genetic difference and geographical distance.** R^2^ = 0.028; *P* = 0.036.

The genetic differences according to the AMOVA results were listed in Table 4. The pairwise Fst values varied from 0 to 1, and most of the pairwise Fst values between populations were significant (*P* < 0.05). The SPSS analysis demonstrated a significant positive relationship between genetic difference and geographic distance (Figure 2).

**Table 4:** **Matrix of pairwise differences (Fst) among the 19 populations calculated by analysis of molecular variance (AMOVA)**

	DR	MQ	QL	DG	JZ	YJ	TK	ZS	SP	HY	BM	TB	ZN	LQ	ZQ	XH	TZ	TD	GD
DR	0																		
MQ	0.19192	0.00000																	
QL	0.30703	0.29714	0.00000																
DG	1.00000	0.68627	0.52787	0.00000															
JZ	0.41905	0.28881	0.26365	0.65143	0.00000														
YJ	1.00000	0.71530	0.32458	1.00000	0.03175	0.00000													
TK	0.46154	0.35667	0.26264	0.74074	0.08374	0.00000	0.00000												
ZS	1.00000	0.65087	0.27098	1.00000	0.04007	0.00000	0.06870	0.00000											
SP	1.00000	0.81274	0.56258	1.00000	0.69817	1.00000	0.75998	1.00000	0.00000										
HY	0.87885	0.43460	0.40379	0.87885	0.46032	0.93529	0.55155	0.91501	0.85957	0.00000									
BM	1.00000	0.77716	0.52787	1.00000	0.65143	1.00000	0.72000	1.00000	0.00000	0.82918	0.00000								
TB	1.00000	0.87597	0.46108	1.00000	0.58503	1.00000	0.63158	1.00000	1.00000	0.96650	1.00000	0.00000							
ZN	0.51515	0.31004	0.24580	0.67347	0.11355	0.51515	0.16579	0.39394	0.73366	0.40043	0.67347	0.51515	0.00000						
LQ	1.00000	0.90289	0.50836	1.00000	0.65147	1.00000	0.69331	1.00000	1.00000	0.97471	1.00000	0.00000	0.61290	0.00000					
ZQ	1.00000	0.80734	0.35664	1.00000	0.43101	1.00000	0.48803	1.00000	1.00000	0.94340	1.00000	0.00000	0.25000	0.00000	0.00000				
XH	1.00000	0.92071	0.38671	1.00000	0.77656	1.00000	0.81081	1.00000	1.00000	0.97740	1.00000	1.00000	0.80247	1.00000	1.00000	0.00000			
TZ	1.00000	0.97706	0.72091	1.00000	0.93564	1.00000	0.94734	1.00000	1.00000	0.99347	1.00000	1.00000	0.95067	1.00000	1.00000	1.00000	0.00000		
TD	1.00000	0.95495	0.46208	1.00000	0.87319	1.00000	0.89480	1.00000	1.00000	0.98717	1.00000	1.00000	0.89565	1.00000	1.00000	1.00000	1.00000	0.00000	
GD	1.00000	0.95495	0.46208	1.00000	0.87319	1.00000	0.89480	1.00000	1.00000	0.98717	1.00000	1.00000	0.89565	1.00000	1.00000	1.00000	1.00000	0.00000	0.00000

### Genetic structure analysis

**Figure 3:**
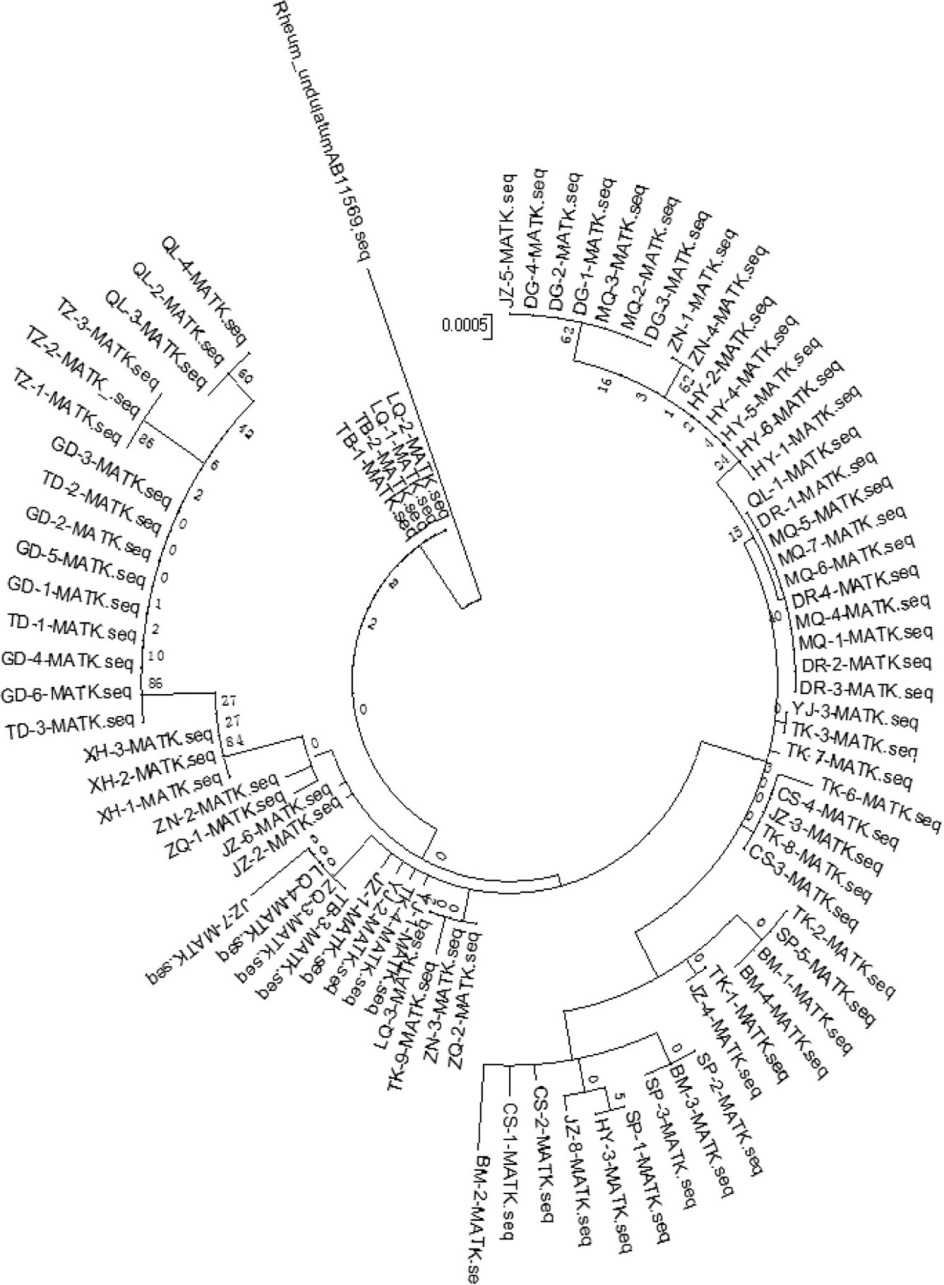
**NJ-tree constructed based on the** ***mat*** **K gene sequences of 87** ***R. tanguticum*** **samples.** *R. undulatum* [GenBank: AB11569] was used as the outgroup.

An NJ-tree was constructed based on the *mat*K gene sequences of 87 *R. tanguticum* samples (Figure 3). The 87 samples were clustered together into two groups, one including the LQ and TB populations, and the other including the remaining 17 populations, which were further clustered into three subgroups. In general, samples from the same population were clustered together, such as the samples from populations QL, TZ, TD, and GD. However, several samples from the same population were clustered into different subgroups, for example, JZ-1, JZ-2, JZ-3, JZ-4, JZ-5, JZ-6, JZ-7, and JZ-8 were all collected from population JZ, but were clustered with different populations.

The results of the NJ-tree analysis were consistent with those of the genetic difference analysis between populations. The genetic differences between populations YJ and TK, SP and BM, DR and MQ, TB and ZQ, and TD and GD were all zero, and these populations were clustered into one subgroup on the NJ-tree. Meanwhile, the genetic differences between populations GD and DR, MQ and TD, and TZ and DG were significant, and they were clustered into different subgroups on the NJ-tree. However, some populations, such as YJ and ZQ, and LQ and YJ, were clustered into the same subgroups on the NJ-tree, but the genetic differences between them were significant (Fst = 1).

### Mismatch distribution analysis

**Figure 4:**
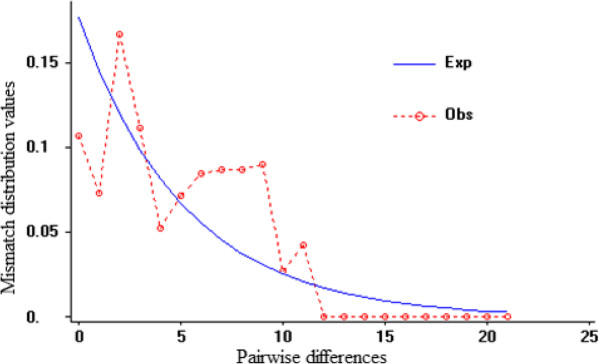
**Mismatch distributions based on the** ***mat*** **K gene sequences of the individual samples.** The straight line represents the expected values and the dotted line represents the observed values.

A mismatch distribution analysis based on Dnasp was performed, and multi-peak traces were obtained to explain the gene exchange present among different populations of *R. tanguticum* (Figure 4). Tajima’s test (Tajima’s D = 1.09761, *P* > 0.10) demonstrated the presence of gene exchange among *R. tanguticum* populations. The average number of migrants (Nm) between populations calculated by AMOVA and Dnasp was 0.05 for both analyses.

## Discussion

In this study, a relatively high genetic diversity was found in *R. tanguticum*, and the genetic diversity among populations was higher than that within populations. Endangered species often showed a relatively low level of genetic diversity [35–38], which was not consistent with this study. In general, many factors were found to influence genetic diversity, such as environmental, genetic, and human factors [39]. *R. tanguticum* is a herbaceous perennial with a long living history [19] and self-incompatible species [23], and its pollen is widely spread from Gansu Province to the Tibet autonomous region in China, *i.e.*, different environmental and climate conditions, thereby enhancing gene exchange and leading to high genetic diversity [40–43].

The distribution of the 15 haplotypes and the SPSS analysis results demonstrated a significant positive relationship between genetic difference and geographic distance. On the NJ-tree, the samples from the same population were clustered together, and the samples from different populations were clustered into different subgroups. Geographic isolation, *e.g.*, by mountains and rivers, was noted among different populations of *R. tanguticum*, and explained why the genetic diversity differed among populations. In this study, the geographic distance between populations JZ and BM was close, but the difference in their haplotypes was significant.

Haplotypes TH1–TH5 were present in different populations at the same time. However, in two populations, JZ and TK, many different haplotypes were simultaneously observed. Although the geographic distances between populations ZS and JZ, DR and QL, and TB and LQ were significant, they had the same genotypes, respectively. On the NJ-tree, some samples from the same population did not cluster into the same subgroup, such as the samples from populations JZ and TK. The genetic differentiation of *R. tanguticum* mainly occurred among different populations. The multi-peak traces and Tajima’s test results (Tajima’s D = 1.09761, *P* > 0.10) demonstrated that the evolution of *R. tanguticum* was consistent with the neutral theory [44], indicating that it did not experience huge environmental changes and rapid expansion. The adaptive capacity to an environment is decided by the genetic diversity of the species, which is also an important index for its long-term survival [45]. As our samples were all collected from untraversed fields without human interference, the gene exchange phenomenon was the result of early accumulation of genetic diversity.

## Conclusion

Genetic diversity among and within populations of *R. tanguticum* in China was demonstrated.

## Abbreviations

SSR: Simple sequence repeats
ISSR: Inter-simple sequence repeats
GST: Coefficient of gene differentiation
NST: Coefficient of gene differentiation taking into account sequence differences
AMOVA: Analysis molecular variance
SPSS: Statistical Product and Service Solutions
Fst: Genetic differentiation values.
